# Supplementary material for: Are carbon emissions trading and green financial instruments synergistic? -Comprehensive quantitative research based on content analysis
Source: PLoS One. 2024 Mar 7;19(3):e0298601. doi: 10.1371/journal.pone.0298601 (PMC10919601; doi:10.1371/journal.pone.0298601)
Supplement: S1 Data — (ZIP) [file pone.0298601.s001.zip › Data/地级市Z_F工作报告.zip!/║╙─╧/╚2├┼╧┐╩╨/└┤╫╘╣1⁄2╓┌║┼í╛┬φ┐╦╩2╛▌═°í┐_2009.docx]

各位代表：

现在，我代表市人民政府向大会作政府工作报告，请予审议，并请各位政协委员提出意见。

一、2008年工作回顾

刚刚过去的2008年，是极不平凡的一年，大事多，难事多，挑战多。市政府在省委、省政府和市委的正确领导下，在市人大、市政协的监督支持下，坚持以党的十七大精神为指导，深入贯彻落实科学发展观，团结带领全市人民，善于借大势发力，勇于在逆境中奋进，努力克服国际国内诸多困难和挑战，沉着应对全球金融危机带来的不利影响，齐心协力，务实苦干，圆满完成了市五届人大三次会议确定的各项任务，经济社会继续保持了平稳较快发展的良好势头。

（一）经济平稳较快增长。预计全年生产总值完成640亿元，比上年增14.5%。地方财政一般预算收入完成36.7亿元，增长25%；一般预算支出完成65.3亿元，增长28.1%，“十件实事”等民生支出得到有效保障。规模以上工业完成增加值346亿元，增长19.5%；实现利润125亿元，增长2%。城镇居民人均可支配收入12370元，增长15%；农民人均纯收入4700元，增长16%。节能减排工作强力推进，关停小火电机组60.4万千瓦，万元GDP能耗下降5.6%；化学需氧量、二氧化硫排放量分别下降13.7%和10%。

（二）项目建设扎实推进。市委、市政府确定的141个重大项目，总投资486.5亿元，截至11月底完成投资154亿元，超额完成年度投资计划。预计全市城镇固定资产投资完成345亿元，增长35%。兰（州）郑（州）长（沙）成品油管道三门峡段完工，郑西铁路客运专线三门峡段基本建成，新三门峡站、市文体中心建设稳步推进，全省首座500千伏输变电工程投入使用；开祥化工100万吨甲醇项目一期等59个项目建成投产，三门峡火电三期1×100万千瓦机组等67个项目前期工作进展顺利。中原黄金冶炼厂、灵宝黄金股份有限公司等4家企业跻身全省百强企业。积极应对金融危机，中央、省项目资金争取工作成绩显著，争取资金3.93亿元，占全省普惠制可争取资金的1/11。

（三）经济结构进一步优化。全年规模以上工业重点项目中，制造、精深加工、高新技术项目比重近1/3。现代服务业规模和效益稳步提升，预计第三产业增加值增长12%。全市文化企业达到1500家，年产值近20亿元；全年接待游客646万人次，实现旅游总收入42.28亿元。非公有制经济增加值占生产总值的46%，成为拉动全市经济的重要力量。消费市场持续活跃，物价涨幅趋于回落，全市消费品零售总额144亿元，增长22.8%。

（四）“三农”工作不断加强。特色农业发展成效明显，预计全年粮食总产61万吨，增长15.5%；苹果总产125万吨，增长14.7%；肉、蛋、奶产量分别达7.1万、4.4万、2.9万吨，增长7%、8%、15%；烟叶收购3.3万吨，创历史新高。新增造林面积66.8万亩，全市森林覆盖率46.7%，荣获“河南省绿化模范城市”称号。亚行高效农业项目全面启动，市级重点农业龙头企业达到28家。我市与河南出入境检验检疫局签署协议，建设全国第一家市级出口果品质量安全示范区。制定农产品国家标准6项，填补了我市无国家标准的空白；康瑞肉牛等16个产品通过国家无公害产品认证，“瑞达牌”鸡蛋等5个农产品被评为河南名牌农产品，仰韶酒被确定为国家地理标志保护产品；陕县张湾乡入选“中国果菜无公害百强乡镇”。新增农村劳动力转移就业3.2万人，实现劳务收入21亿元；完成搬迁扶贫820户3410人；解决了12万农村居民的饮水安全问题。新农村建设分类推进，464个重点村基本实现年度建设目标。

（五）城镇化水平逐步提高。城市引水工程克服重重困难胜利竣工，市区居民吃上了甘甜的山泉水。“城中村”改造一期工程完成招投标，旧城改造指导意见制定出台。黄河西路、虢国东路改造基本完成，大岭路涧河大桥进展顺利。城区电网设施不断完善，逐步实现入地运行。天鹅湖城市湿地公园被批准为国家湿地公园。园林城市创建通过省级复检，卫生城市、环保模范城市、文明城市创建工作完成年度目标。义（马）渑（池）城区快速通道建设进展顺利，全市城镇化率达43.9%，提升1.5个百分点。县域经济快速发展，5个县（市）中3个进入全省综合实力20强，4个进入财政收入30强。

（六）改革开放不断深入。强力推进重点领域和关键环节的改革，中原黄金冶炼厂成功实现战略重组，义煤集团股份有限公司揭牌成立，城市信用社组建商业银行获得国家银监会批复，建设、林业、水利、交通、文化等领域的改革扎实推进。旅游节和中博会共签约5000万元以上项目103个，签约金额418.5亿元，项目落地率80.3%。为抵御全球金融危机的不利影响，我们坚持“危”中求“机”，主动承接国际国内产业转移，成功举办无锡和北京项目推介会，共签订投资合同和意向38个，投资金额138.6亿元。预计全年实际利用外资2.34亿美元，增长25.1%。金融生态环境不断优化，荣获“中国十佳新锐金融生态城市”和“中国金融生态城市”称号，银企合作大会签约合同和意向118个，金额131.64亿元。对外友好交流工作取得突破性进展，与美国、法国、匈牙利、韩国和澳大利亚等国的友好联系不断加强。“黄河金三角”区域协调发展综合试验区建设开始启动。中国海关、中国商检驻三门峡办事处和国家铝及铝制品检测中心建设前期工作扎实推进。

（七）和谐社会建设取得成效。省、市“十件实事”完成或达到进度计划。新增城镇就业5.6万人，下岗失业人员实现再就业2.3万人，城镇登记失业率3.4%；企业退休人员养老金人均增长104元。城镇居民医疗保险制度全面实施；新建农村敬老院9所；完成棚户区改造100万平方米；开工廉租房2.5万平方米。科技贡献率逐步提高，高新技术产业实现产值61.2亿元，增长11.3%。高等教育实现跨越式发展，三门峡职业技术学院分别与匈牙利索尔诺克大学和美国得州大学达成合作办学协议；农村中小学校舍改造完成3.7万平方米，市区阳光小学教学楼主体封顶。市中心医院病房楼开工建设，建成农村甲级卫生室30个。新建“农民体育健身工程”80个，被国家体育总局授予“全民健身与奥运同行活动优秀组织奖”。人口和计划生育工作取得新进展，人口自然增长率控制在4.5‰以内。卢氏灾后重建扎实推进，受灾群众生产生活条件逐步改善；全力支援四川抗震救灾，圆满完成抢险救援和过渡房建设任务，对口援建工作走在全省前列。创新信访和群众工作机制，义马经验、渑池模式在全国推广，深入开展“大接访、大下访、大包案、大解决”活动，一大批信访积案得到有效化解。大力开展“平安三门峡”建设，严厉打击刑事犯罪，城区技防监控系统建成，社会治安综合治理不断强化，群众安全感持续提升。严格落实安全生产责任制，加强全民安全教育，强化煤矿等重点领域的安全管理，安全生产形势稳定。加强食品安全监管，妥善处置“三鹿奶粉”和“山西毒羊肉”事件，食品放心工程建设扎实推进。

（八）精神文明和民主法制建设扎实推进。精神文明创建工作成效显著，中国楹联文化城市创建取得新进展。扩大基层民主，第六届村委会换届选举平稳完成。坚持依法行政，自觉接受市人大及其常委会的监督，支持政协参政议政，办理人大代表建议90件、政协委员提案282件，满意和基本满意率均达到100%。深入开展“廉政建设年”活动，廉政建设和反腐败斗争取得新成效。国防教育和双拥工作进一步加强，民兵预备役工作有了新提高，驻市解放军和武警部队在抗震救灾、维护社会稳定、支持地方建设等方面作出了突出贡献。市工商联荣获全国工商联系统先进单位。统计、审计、物价、粮食、侨务、对台、老龄、档案、史志、人防、气象、无线电、民族宗教、妇女儿童、河务移民、残疾人等工作都取得了新成绩。

过去一年的成绩，是在国内外环境复杂多变、约束因素不断增加、区域竞争日益加剧的情况下取得的，的确来之不易，经验十分珍贵。认真回顾总结，主要有以下五点体会：一是坚持解放思想。紧紧抓住解放思想这个总开关，认真开展“新解放、新跨越、新崛起”和“解放思想，科学发展，奋力实现新跨越”大讨论活动，努力提高贯彻落实科学发展观的自觉性和坚定性，扭住经济建设这个中心不放松，坚定不移地推进改革开放，统筹兼顾做好各项工作，努力实现经济社会平稳较快发展。二是积极应对挑战。面对困难局面，既清醒认识到全球金融危机造成的冲击，增强忧患意识，又辩证地看到“危”中有“机”，“危”后有“机”，抓住机遇，借机发力，扎实推进项目建设，不断加快结构调整，加快发展方式转变。三是密切关注民生。在努力保持经济平稳较快发展的同时，更加注重以省、市“十件实事”为重点的民生工程建设，着力解决上学就医、劳动就业、社会保障、生活改善等涉及群众利益的热点、难点问题，切实维护社会和谐稳定。四是优化发展环境。围绕信用、金融、服务环境建设，深入开展行风和机关作风效能评议，强化服务意识，提升服务水平，为经济发展营造了良好的环境。五是狠抓工作落实。以“两转两提”为重点，不断加强政府自身建设，大力倡导“严、细、深、实”的工作作风，抓深入、抓具体、抓落实，为促进经济社会平稳较快发展提供了有力保障。

过去一年的成绩和经验，是在省委、省政府和市委的正确领导下，全市人民团结奋进、克难攻坚的结果，凝聚着社会各界人士的心血和智慧。值此，我代表市人民政府，向全市广大工人、农民、知识分子、干部、人民解放军指战员、武警官兵、公安民警以及社会各界人士致以崇高的敬意和衷心的感谢！

同时，我们也清醒地看到，政府工作与人民的期待还有一定差距，前进道路上还存在许多不容忽视的困难和问题。一是受国际金融危机的影响，保持经济平稳较快增长的难度加大；二是产业结构不够合理，工业层次较低，产品链条短，特色农业效益不高，第三产业发展滞后；三是发展方式粗放，依法依规利用土地意识不强，节能减排和环境保护压力较大；四是城乡公益事业和基础设施欠账较多，建设任务非常艰巨；五是政府职能和作风转变还不到位，个别部门和单位的大局意识、服务意识不强，作风疲沓，形式主义、官僚主义问题依然存在。对此，我们一定以更加开放的思维，更加开阔的眼界，更加务实的作风，更加奋力的苦干，有针对性地采取措施，认真加以解决。

二、2009年政府工作总体要求和奋斗目标

今年是新世纪以来我国经济发展极为困难的一年，也是蕴含重大机遇的一年。积极应对国际金融危机严重冲击，扎实做好今年的工作，对于维护我市改革发展大局，加快全面建设小康社会进程、跻身中原崛起第一梯队具有十分重要的意义。虽然我们面临着来自国际国内的严峻挑战，但全国和省、市经济发展趋势没有改变，重要战略机遇期依然存在。经过这些年的积累和发展，我市支柱产业不断壮大，经济实力显著增强，抗御风险的能力逐步提高，市场运作的经验日渐丰富，全市上下干事创业的热情空前高涨，特别是去年项目资金争取工作首战告捷，实施“走出去”战略、加大招商引资力度、主动承接国内外产业转移成效显著，必将对拉动内需、促进增长起到有力的推动作用。当前我们遇到的问题是发展中的问题，面临的困难是前进中的困难，只要全市上下同心同德，团结一致，奋发有为，扎实工作，我们一定能够战胜困难，渡过难关，夺取今年经济社会发展的新胜利！

政府工作的总体要求是：全面贯彻党的十七大和十七届三中全会精神，深入贯彻落实科学发展观，紧紧围绕跻身中原崛起第一梯队的奋斗目标，扩内需、保增长、调结构、促转型，立足保持经济平稳较快增长，切实加快经济结构调整和发展方式转变，积极推进改革开放和体制机制创新，下更大力气改善民生、促进社会和谐，审时度势抓机遇，振奋精神促发展，奋力开创三门峡经济社会跨越式发展新局面。

经济社会发展的主要预期目标是：全市生产总值增长11%左右。地方财政一般预算收入增长11%左右。全社会固定资产投资增长21%。社会消费品零售总额增长15%。外贸进出口增长10%；实际利用外资增长13%。居民消费价格涨幅控制在4%左右。城镇居民人均可支配收入增长9%；农民人均纯收入增长7%。人口自然增长率控制在6.5‰以内。城镇登记失业率控制在4.6%以内。节能减排完成省定目标。

三、全力以赴应对金融危机，努力保持经济平稳较快发展

（一）在危机中抢抓机遇，全力争取项目资金。强力实施“8155”投资促进计划。扎实开展“项目建设年”活动，以农林水、交通、能源、城镇建设、自主创新、产业升级、节能减排、社会事业8个方面为投资重点，实施100个投资上亿元的项目，新开工项目的总投资超过500亿元，力争当年全社会固定资产投资达到500亿元。切实加强项目衔接。围绕实施“8155”计划，加强领导，明确责任，抽调精干力量，积极做好项目沟通衔接，力争多纳入国家、省重点项目盘子，争取上级在资金、建设用地及项目审核方面给予更大的支持。全面抓好项目落实。积极落实配套资金，加大财政投入力度，拓宽融资渠道，拉动社会投资。认真落实好项目用地，全面做好项目建设服务，确保项目如期开工。加快在建项目建设进度，确保尽快发挥效益。

（二）强力推进重大基础设施建设，努力扩大投资需求。加快交通运输体系建设。加快郑西客运专线新三门峡站站房楼、站前广场建设，尽快开工新车站到金昌路立交桥的迎宾大道建设，确保实现同步投运。加大工作力度，力争连霍高速三门峡段拓宽工程、运三黄河公铁特大桥等重大项目年内开工，争取三（门峡）淅（川）高速公路、运（城）三（门峡）铁路早日开工，构建“三纵四横”大交通网络，建设黄河“金三角”地区交通枢纽中心。抓好民生项目建设。加快廉租住房建设步伐，新开工廉租住房2万平方米，竣工1万平方米；全面完成棚户区改造任务。推进农业、水利等基础设施工程，完成灵宝窄口等20个水库除险加固工程年度任务，加快渑池青莲河等5个水土流失综合治理项目建设。新建和改造县乡公路260公里、通村公路150公里，解决8万人饮水安全问题，新发展沼气用户2万户，建成大、中型沼气项目3座，新建100个农村连锁超市。推进电气设施建设。加快电网建设，确保灵宝换流站扩建工程主体完工，开工建设三门峡东部500千伏输变电工程。做好国家“西气东输”二线建设前期工作，争取早日开工，抓好我市分供站的规划衔接工作。

（三）强化经济运行调节，优化经济发展环境。建立健全经济运行协调机制。完善经济运行监测预警系统，加强对重点行业、企业的监测。加强煤、电、油、运等要素的综合协调，建立煤炭和电力企业的合作机制，保障电煤供应。积极推进大户用电直供试点，实现电力企业与用电大户合作共赢。在工业系统开展“质量效益管理年”活动，全面提升企业管理水平。继续落实市领导分包重点企业制度，帮助企业解决发展难题。抓好财政金融工作。完善税收征管机制，规范非税收入管理，促进财政经济协调增长。从严控制一般性支出，优先保证重点支出和法定支出，确保改善民生、落实配套资金、办好实事等资金需要。以财政资金为引导，鼓励社会资本投资成立各类担保机构，建立市、县担保体系。支持大型骨干企业和政府投融资主体发行债券，支持符合条件的企业发行短期融资券。加强企业和个人征信体系建设，进一步优化金融生态环境。抓住国家实行适度宽松货币政策的机遇，鼓励和引导金融机构全面落实金融促进经济发展的政策措施，争取国家拉动内需项目的银行配套贷款投入。开展多种形式的银企洽谈会，组织关联度高的企业开展产业链融资，缓解企业资金困难。提升国土资源保障能力。认真落实好耕地保护和节约集约用地制度。继续推进新一轮土地利用总体规划修编，全面加强土地利用的规划控制；科学合理配置用地指标，优先保障省、市重点项目用地需求；进一步扩大多层标准厂房使用范围，建立完善砖瓦窑场整治长效机制和节约集约用地考核评价管理约束机制。深入推进矿产资源整合，提高资源利用效率，加大未探明矿产资源的勘探力度，优先配置给优势龙头企业。进一步优化经济发展环境。高度关注企业生产运行面临的困难，帮助和支持企业渡过难关。依法清理各种涉企收费和基金项目，严厉查处“四乱”以及危害项目建设和企业经营的违法犯罪行为，营造严明的法制环境。继续推进“信用三门峡”建设，以良好的环境吸引客商、留住客商。

四、调整优化产业结构，转变经济发展方式

（一）加快产业结构调整，不断优化经济结构。继续壮大支柱产业。围绕五大支柱产业，拉长产品链条，打造产业集群，提升核心竞争力。能源方面，鼓励煤电企业联营，建设大唐公司1×100万千瓦机组和风电二期、渑池热电联产等项目；铝工业方面，继续开发汽车零部件、工业铝材等高科技项目，建设戴卡公司200万只轿车轮毂、中迈铝业30万吨高精度铝板带箔等项目，通过铝的精深加工实现增值；煤化工方面，依托义马气化厂、开祥化工现有产能，建设开祥化工100万吨甲醇二期，义煤集团与美国SES公司合作1000万标方煤制气、30万吨醋酸等项目，加大下游产品的开发；黄金及有色工业方面，加大地质找矿力度，加强黄金伴生金属开发，建设灵宝华鑫公司1万吨铜箔、灵宝华宝公司20万吨铅冶炼等项目；林果业方面，围绕终端消费品生产，建设灵宝景源果业2万吨浓缩葡萄汁、阿姆斯公司3万吨石榴汁等项目。推动高新技术产业化和制造业发展。以新材料、电子信息等领域为重点，集中支持煤化工、铝精深加工等关键技术研发及产业化，加快企业研发中心建设，提升企业技术创新能力。以精密量仪、成套设备为重点，加快装备制造业发展，开工建设宏达公司数控煤矿机床、新华水工机械公司大型成套设备等项目。发展节能照明产业，建成鹏飞公司100万只无极灯项目。加快工业企业战略重组。进一步强化政府引导，因企制宜，分类指导，突出重点行业和领域，加快煤及煤化工、铝工业、黄金、果品加工等关联企业的战略重组和合作步伐。鼓励龙头企业和重点企业通过互相参股控股，扩大产业规模，提高产业集中度，提升优势产业抗风险能力。加快产业聚集区建设。抓住省重点支持的第一批7个产业聚集区，加快区内供排水、污水垃圾处理、道路等基础设施建设。力争三门峡工业园天瑞30万吨铝铸件等6个项目建成投产，促使华联创业园等4个项目开工建设。借鉴重庆、大连等地的经验，制定优惠政策，鼓励市区工业企业向工业聚集区搬迁，促进产业集聚发展。大力发展服务业。认真落实加快服务业发展的指导意见和服务业发展规划，依托资源优势和现有产业基础，改造提升传统生活性服务业，加快发展生产性服务业，重点推进物流、商贸、旅游产业发展，全面提高服务业水平。加快大鹏酒店招商引资步伐，引进家乐福超市等国内外知名连锁企业入驻，支持七海物流公司发展，力争建成九州通仓储加工配送中心，开工建设三门峡市物流配送中心。发展壮大文化产业，开工建设虢国博物馆二期工程、渑池仰韶博物馆，力争虢国文化产业园、函谷关老子文化园、仰韶文化产业园成功申报省级文化产业园区。启动灵宝函谷关等旅游项目改造，抓好精品景区建设，新增4A级景区1个。开工建设上阳苑仿唐文化一条街和卢氏游客服务中心。加强旅游宣传推介，强力开拓旅游市场，扩大旅游品牌的影响力。加快金融业发展，做好城市商业银行挂牌运行和各类金融保险机构的引进工作。

（二）扎实推进节能减排，建设资源节约和环境友好型社会。强力推进节能降耗。突出抓好工业、交通、建筑等关键领域的节能工作，加快淘汰落后产能。严格项目节能评审，严把新建项目能耗准入关。加强电力、煤炭、有色、建材、化工等重点行业节能降耗，推进东方希望节能改造、锦荣水泥余热发电等一批节能工程建设。加强环境保护和生态建设。严格实施环境影响评价制度、“三同时”制度、排污许可证制度、问责制和“一票否决”制。加强市、县两级环境监控系统平台建设，完成城市污水处理厂COD和氨氮自动监控设备更新改造。确保污水处理厂全面稳定达标运营，燃煤电厂脱硫设施投运率达到95%以上。继续抓好林业生态三级联创，加快城区大环境绿化和沿黄生态林建设，完成植树造林56.9万亩，其中退耕还林4万亩。加强农村环保工作，全面实施农村小康环保行动计划，建成3个环境优美小城镇和14个省级生态文明村。建立健全节约资源、保护环境的长效机制。积极培育低碳经济和循环经济，大力发展节能环保产业，重点建设一批循环经济试点园区和试点企业。推广水环境生态补偿制度，完善排污收费机制，对排污大户适当提高排污费、污水和垃圾处理费征收标准。积极争取并用好上级节能奖励资金，对城市污水处理、垃圾处理、农村环境整治实行先建后补。深入开展全民节能减排行动，积极倡导节约型生产方式、消费模式和生活习惯。

五、加快推进城乡建设，统筹城乡协调发展

（一）切实抓好“三农”工作，加快农村改革发展。认真落实好各项支农惠农政策。大幅度增加对“三农”的投入，保证各级财政对农业投入增长幅度高于经常性收入增长幅度；增加对农村基础设施建设和社会事业发展的投入，提高政府土地出让收益、耕地占用税新增收入用于农业的比例，增加农村公益性建设项目的投入。大幅度增加农资综合直补、粮食直补、良种补贴和农机具购置补贴等规模；扩大“家电下乡”补贴品种范围；落实支持振兴奶业和稳定生猪生产财税扶持政策。大力发展特色农业。围绕调优一产，壮大特色支柱产业，突出抓好豫西优势林果、310国道沿线畜牧、三门峡优质烟叶、沿黄蔬菜、豫西食用菌和西南部山区中药材六大特色农业产业区，建设特色农业强市。围绕出口果品质量安全示范区建设，新建苹果出口基地10万亩，努力建成全国重要的果品生产、加工、出口基地；新建和改建标准化规模养殖场30个，标准化养殖小区20个，万吨以上畜产品加工企业3—5个；烟叶面积稳定在25万亩左右，着力提高产量、质量，提高经济效益，争创全省烟叶生产第一大市。继续推进菌、药等产业发展，依托渑池万亩玫瑰园建设，推动花卉园艺及深加工产业发展。分类推进新农村建设。按照“一乡一业”、“一村一品”的发展思路，繁荣农村经济，增加农民收入。扎实推进村容村貌整治，完成454个村庄建设整治任务，力争2010年全部达到一类村庄上水平、二类村庄上台阶、三类村庄变面貌的总体目标。继续抓好试点村领导包扶工作，动员和鼓励回乡农民参与新农村建设。继续开展“红旗渠精神杯”竞赛活动，加大“四荒”开发力度。继续抓好搬迁扶贫，确保完成750户3100人的安置任务。完善农村服务体系。加快农业产业化、标准化、信息化步伐，大力扶持农业龙头企业，力争年产值超亿元的龙头企业达到10家以上，完善农产品质量检测体系，确保农业信息化继续走在全国前列。大力发展农民专业合作组织，确保农民专业合作社达到200家，带动农户60%以上。加强新型农业科技推广体系建设，构建农业科技推广新机制。加大农机新技术、新机具引进推广力度，提高农业装备水平。加大培训转移力度，确保全年转移农村劳动力28万人次，实现劳务收入15亿元。

（二）抓好中心城市建设，进一步加快城镇化进程。完善中心城市功能。加强城市基础设施建设，确保大岭路涧河大桥投入使用，崤山路改建全面完工。健全城市公共服务设施，建成市儿童福利院，开工建设市文体中心，加快市广电中心前期工作。加快推进台上区集中供热，稳步建设城市燃气网络和城市电网。强力推进“城中村”改造，确保一期工程开工，全面启动旧城改造。年内通过国家园林城市验收，争取早日实现“四城联创”目标。努力改善城市人居环境。加快天鹅湖国家城市湿地公园建设步伐，抓好涧南公园、涧河北岸绿地和虢国公园二期改造，加快重点路段和公园景区的亮化、美化，提升城市形象。改进和加强城市管理，营造便民、利民、惠民的人居环境。加快副中心城市和小城镇建设。继续推进义马—渑池城区对接，确保义渑快速通道义马段竣工通车；开工建设三门峡至灵宝快速通道，带动沿路城镇产业带发展。大力发展县域经济，突出复位晋位，不断增强县域综合实力。总结义马城乡一体化试点经验，以城乡统筹、协调发展为主要内容，推进全市城乡一体化。支持故县、大王、观音堂、英豪、五里川、杜关等重点镇加快发展，引导经济要素向城镇集聚，切实发挥小城镇在经济、文化等方面的辐射带动功能。

六、高度关注民生，促进社会和谐

（一）努力扩大社会就业。实施更加积极的就业政策，确保全年城镇新增就业4万人，实现下岗失业人员再就业1.3万人。发挥政府投资、公益性岗位对稳定就业的导向作用，保护和提高劳动密集型产业、中小企业和服务业吸纳就业的能力。综合运用财政、金融、税费减免等优惠政策，通过全民创业带动就业。发挥好政府投资和重大项目带动就业作用，努力提供就业机会。用好就业专项资金和失业保险基金，进一步开辟面向就业困难群体的公共管理服务岗位，动态消除城镇“零就业家庭”。安排农民工创业专项扶持资金，继续实施“阳光工程”和“雨露计划”，认真做好返乡农民工就业工作，积极引导农民向二、三产业转移，拓展农民就业空间。加大对高校毕业生就业援助工作力度，鼓励他们到基层和中小企业工作。支持困难企业采取在岗培训、轮班工作、协商定薪等办法，尽量不裁员或少裁员。

（二）进一步完善社会保障体系。继续做实企业职工基本养老保险个人账户，积极推进城镇企业职工养老保险省级统筹。继续增加企业退休人员基本养老金，人均增长10%左右；提高企业退休人员取暖补贴标准，由年人均30元提高到480元。完善失业保险制度，确保失业保险金按时足额支付。逐步将失地农民纳入就业和社会保障体系。加快完善基本医疗保障制度，鼓励城乡就业人员按规定参加城镇职工医保。继续加强城镇居民医疗保障，适当扩大报销范围、提高报销比例，将在校大学生全部纳入城镇居民医保范围，引导灵活就业人员自愿参加城镇职工或居民医保。进一步做好新型农村合作医疗工作，提高农村医疗保障水平。落实城市居民最低生活保障制度，实现动态管理下的应保尽保；进一步完善农村低保，逐步提高补差标准。加快乡镇敬老院建设，确保集中供养率达到45%。

（三）大力发展各项社会事业。整合城区教育资源，规划建设市职业教育园区；建成阳光小学，开工建设三门峡外国语高中；实施农村初中校舍改造，改善农村办学条件；认真解决农民工子女在流入地免费接受义务教育问题；推进教育人事制度改革；促进高等教育、职业教育、成人教育协调发展。完善科技服务体系，加快科技成果转化。加强卫生基础设施建设，确保三门峡市中心医院病房大楼主体完工，改建12所乡（镇）卫生院、4家社区卫生服务站，建设300所标准化村级卫生室。免费实施白内障康复手术3000例，建成“白内障无障碍市”。深化文化体制改革，加强农村公共文化服务体系建设，开工20个乡（镇）综合文化站，完成豫西文化长廊重点区域建设任务。加强文物保护和利用，扎实做好丝绸之路“申遗”和大遗址保护工作。深入开展群众性体育活动，建成87个农民体育健身工程。继续做好人口和计划生育工作，农村计划生育家庭奖励扶助标准由600元提高到840元；继续在全市农村实行住院分娩补助政策，为农村孕妇免费补充叶酸，提高出生人口质量。扎实推进对口支援四川江油灾后重建工作。

（四）深入开展精神文明创建活动。加强社会主义核心价值观体系建设，大力弘扬民族优秀文化传统。着力提高市民文明素质和城市文明程度，开展以优美环境、优良秩序、优质服务，创建文明城市、文明社区、文明景区为内容的“三优三创”竞赛活动。推进社会文化环境净化工程，加强大学生思想政治工作和未成年人思想道德建设。加强农村精神文明建设，建设一批文明村镇。

（五）全力维护社会和谐稳定。深入开展整顿和规范市场秩序专项行动以及“质量和安全年”活动，切实加强食品药品安全监管。进一步强化煤矿、非煤矿山、化工、道路交通、消防等重点行业的安全治理，落实各项整改措施，坚决遏制重特大安全事故发生。加强群众工作，着力解决信访突出问题，高度重视和有效防范股市房市、企业破产、征地拆迁、农村土地纠纷、涉法涉诉等可能引发的群体性事件。深入开展平安创建活动，加强社会治安综合治理，依法打击各类犯罪行为，增强人民群众的安全感。充分发挥工会、共青团、妇联等人民团体密切联系群众的桥梁纽带作用，积极参与社会事务管理。继续做好国防动员和人民防空工作，加强全民国防教育和民兵预备役建设，积极开展双拥共建活动，巩固和发展军政、军民关系。重视发展民族宗教、外侨、对台、史志等各项事业，加强气象服务、防震减灾工作。

七、继续坚持解放思想，强力推进改革开放

（一）扎实推进各项改革。继续深化财税管理制度和预算制度改革，支持银行、保险、证券机构的改革发展。推进投资体制改革，健全政府投资管理体制。加快水管体制改革，3月底前完成验收。不断深化农村改革，在家庭承包经营的基础上，加快农村土地流转，发展多种形式的适度规模经营；改善和创新农村金融服务，解决农民贷款难问题；全面推进集体林权制度改革。努力解决国企改革的遗留问题。

（二）进一步扩大对外开放。充分发挥招商引资的带动作用。创新招商引资方式，积极开展针对性招商，瞄准国内外重点企业，引进战略投资者。围绕招商引资和拉动内需，创新办节模式，办好第十五届三门峡国际黄河旅游节暨投资贸易洽谈会。优化进出口产品结构，扩大对外贸易规模，用好国家出口退税政策，支持纺织等劳动密集型产品出口；积极争取出口信贷支持，扩大机电产品和高新技术产品出口；扩大紧缺资源、先进技术、关键零部件进口。加快推动中国海关、中国商检驻三门峡办事处和国家铝及铝制品检测中心筹建，建立“大通关”机制。继续深化区域经济合作。以建设“黄河金三角”区域协调发展综合试验区为契机，加强与运城市、渭南市、临汾市的合作交流，深化资源开发与产业发展合作，实现资源共享、信息互通、合作多赢。鼓励与沿海发达地区建立产业转移联盟，设立产业转移承接园区，承接电子、家电、服装、家具等产业的转移。

（三）全面激发非公有制经济活力。认真贯彻落实进一步促进全民创业、加快非公有制经济发展的有关措施，继续坚持“放心、放胆、放手、放宽、放活”的方针，鼓励、支持和引导非公有制经济发展。设立中小企业发展专项资金，完善促进中小企业发展的体制机制。按照市场化运作方式，选择一批有规模、有市场、有发展前景的中小企业予以重点扶持，促进企业发展壮大。

八、深入开展“两转两提”，加强政府自身建设

（一）更加注重职能转变和管理创新，努力建设服务政府。继续转变政府职能，加强制度建设，健全行政决策、执行、咨询、反馈、监督等方面的程序和制度。深化行政管理体制改革，加快推进政企分开、政资分开、政事分开、政府与市场中介组织分开，加强宏观经济调控，减少对微观经济的直接干预。积极稳妥地推进新一轮机构改革，上半年完成市政府机构改革，年底前完成县级政府机构改革。继续做好事业单位分类改革。深化行政审批制度改革，继续精简市级行政审批项目，规范政府审批和行政许可行为。改革行政服务工作机制，健全岗位责任制、服务承诺制、限时办结制、首问负责制、效能考评制和失职追究制。加强市、县、乡三级行政便民服务网络建设，增强服务意识，精简办事程序，提高工作效率。继续做好应急工作，不断完善应急体系。

（二）更加注重依法行政和民主法制建设，努力建设法治政府。坚持依法行政，理顺执法体制，强化执法责任，推进文明执法。严格执行人大及其常委会的决议、决定，自觉接受人大及其常委会的法律监督和工作监督。自觉接受人民政协的民主监督。广泛听取各民主党派、工商联和无党派人士的意见和建议。支持监察、审计等部门依法独立履行职责。依照有关法律的规定，接受司法机关的监督。重视发挥新闻舆论和社会公众的监督作用。认真实施《政府信息公开条例》，大力推广电子政务和网上办公。不断优化政府办事规程和行为准则，健全以行政首长为重点的行政问责制度，加大责任追究力度。

（三）更加注重廉政建设和反腐败工作，努力建设廉洁政府。落实党风廉政建设责任制，着力完善惩治和预防腐败体系，认真执行领导干部廉洁自律规定，强化权力运行制约和监督。各级领导干部和政府工作人员都要做到廉洁自律、自警自励，做遵纪守法的模范、廉洁奉公的表率。牢固树立过紧日子的思想，对公务购车用车、会议经费、公务接待以及出国出境考察等支出实行“零增长”，严格控制新建楼堂馆所，坚决制止铺张浪费、奢靡之风，做到令行禁止、违者必究。

（四）更加注重作风转变和能力提升，努力建设效能政府。各级政府要深入开展科学发展观学习实践活动，扎实推进“两转两提”，切实提高政府公信力、执行力。在应对金融危机的关键时期，广大政府工作人员要励精图治，振奋精神，增强信心，迎难而上，形成加快发展、共克时艰的强大合力。发扬求真务实和艰苦奋斗精神，多干打基础、利长远的事，多抓加强基层基础工作的事，特别要继续办好省、市“十件实事”，努力作出经得起实践、人民和历史检验的实绩。坚持深入实际、深入基层、深入群众，开展调查研究，做到问政于民、问需于民、问计于民。深化机关作风效能评议活动，切实改进会风、文风，大力精简会议、文件，坚决反对虚报浮夸、弄虚作假，坚决反对官僚主义、形式主义。树立正确的政绩观，更加紧迫地学习新知识、增长新本领，着力把握发展规律，创新发展理念，切实提高为基层、为群众服务的能力和水平。

各位代表，2009年必定是三门峡人民不畏艰难、奋发进取、共克时艰的一年，困难和挑战在考验着我们，使命与希望在激励着我们，让我们在省委、省政府和市委的正确领导下，团结拼搏，迎难而上，为促进经济社会平稳较快发展、跻身中原崛起第一梯队而努力奋斗！
